# Complete plastome and mitogenome assembly of endangered tree *Karpatiosorbus bristoliensis* reveals phylogenetic architecture for *Sorbus* sensu lato (Rosaceae)

**DOI:** 10.3389/fpls.2025.1619267

**Published:** 2025-07-08

**Authors:** Qiang Li, Ran Wei

**Affiliations:** ^1^ College of Life Sciences, Nanjing Forestry University, Nanjing, Jiangsu, China; ^2^ College of Manufacturing Engineering, Maanshan University, Maanshan, China

**Keywords:** *Karpatiosorbus bristoliensis*, *Sorbus* s.l., mitochondrial genome, RNA editing events, gene transfer, phylogenetic analysis

## Abstract

**Introduction:**

The genus *Sorbus* sensu lato (*Sorbus* s.l.) comprises over 260 species widely distributed across temperate regions of Asia, Europe, and North America. However, hybridization and polyploidization have posed significant challenges to phylogenetic and taxonomic studies within this genus.

**Methods:**

Here, we assemble the first complete chloroplast and mitochondrial genomes of *Karpatiosorbus bristoliensis* to characterize organellar genomic features and establish a maternally inherited phylogenetic framework for *Sorbus* s.l.

**Results and discussion:**

The mitochondrial genome of *K. bristoliensis* is circular (386,757 bp), encoding 55 genes, including 34 protein-coding genes, 18 rRNAs, and 3 tRNAs. Its chloroplast genome has a typical quadripartite structure (160,322 bp), containing 75 protein-coding genes, 29 tRNA genes, 4 rRNA genes, and one pseudogene (*ycf1Ψ*). Homologous gene transfer analysis of *Sorbus* s.l. species revealed inter-organellar gene transfer ranging from 3,021 to 3,846 bp. RNA editing analysis revealed 274–352 editing sites in *Sorbus* s.l., with *nad4* containing the greatest number of editing sites across all protein-coding genes except those in *K. bristoliensis*. Simple sequence repeat (SSR) analysis detected 48–52 SSRs per species, predominantly mononucleotide repeats. Phylogenetic reconstruction on the basis of organellar genomes revealed that *Karpatiosorbus* is a sister to *Torminalis*. Plastome-based phylogeny revealed the non-monophyletic status of *Sorbus* s.s., attributed to the nested placement of the hybrid-origin genera *Hedlundia* and *Scandosorbus* within the genus. Additionally, *Hedlundia austriaca* and *H. persica* should be transferred to the nothogenus *Sorbomeles*. Mitochondrial genome collinearity analysis revealed extensive genomic structural rearrangements. Our findings not only delineate the structural characteristics of mitochondrial genomes across *Sorbus* s.l. taxa but also establish a high-resolution maternal phylogenetic framework for this genus.

## Introduction

The genus *Sorbus* L. were primarily established by Linnaeus and included only two species (*S. aucuparia* L. and *S. domestica* L.) (Linnaeus, 1753). Subsequently, more species were transferred to this genus. *Sorbus* L. broadly (*Sorbus* L. sensu lato) contains approximately 260 species characterized by both simple-leaved species and pinnately compound-leaved species (Yü and Lu, 1974; Phipps et al., 1990; Lu and Stephen, 2003). *Sorbus* s.l. comprises trees or shrubs typically restricted to high altitudes, occupying diverse habitats including mountain valleys, streamsides, rocky slopes, and scrublands (Lu and Stephen, 2003). These plants are widely distributed across temperate Asia, Europe, and North America (Phipps et al., 1990). Many species exhibit ornamental value due to their conspicuous colorful fruits (McAllister, 2005).

Previous molecular and morphological evidence suggests that *Sorbus* s.l. is paraphyletic and raises six subgenera to the generic rank (*Aria* (Pers.) Host, *Cormus* Spach*, Chamaemespilus* Medikus*, Micromeles* Decaisne, *Sorbus* sensu stricto (*Sorbus* s.s.), and *Torminalis* Medikus) (Campbell et al., 2007; Zheng and Zhang, 2007; Potter et al., 2007; Lo and Donoghue, 2012). Among the six genera, the genera *Aria*, *Chamaemespilus*, *Micromeles*, and *Torminalis* all have simple leaves, whereas *Cormus* and *Sorbus* s.s. are characterized by compound leaves. However, the generic boundaries of these taxa have been continuously revised. For instance, Mezhenska et al. (2018) proposed transferring all Asian simple-leaved species to the genus *Micromeles*, including the previously taxonomically disputed genera *Aria* and *Micromeles*. In the same year, Rushforth (2018) alternatively classified Asian simple-leaved species within *Micromeles* and five newly established genera (*Griffitharia, Alniaria, Thomsonaria, Dunnaria, and Wilsonaria*) on the basis of morphological characteristics.

The primary factors contributing to the taxonomic complexity within *Sorbus* s.l. are hybridization and polyploidization. Notably, previous works highlighted that polyploidization, frequent natural hybridization and apomixis play crucial roles in the ongoing genetic diversification of *Sorbus* s.l. species (Robertson et al., 2010). Biparental, triparental and multiple-hybrid origins (Németh et al., 2020; Nelson-Jones et al., 2002; Robertson et al., 2004) contribute to allodiploid, triploid and tetraploid species, rendering the taxonomy complex. In addition, interspecific hybridization also promotes the innovation of morphological characteristics, such as the number of pairs of leaflets and fruit color (Nelson-Jones et al., 2002; Wu et al., 2024).

The taxonomic treatment of numerous hybrid-origin species remains unresolved, particularly with respect to their delimitation and placement within phylogenetic frameworks. These hybrid-origin species have been taxonomically elevated to subgenera (Rich et al., 2014) or genera (Sennikov and Kurtto, 2017). For example, Sennikov and Kurtto (2017) elevated certain hybrid-origin taxa within the genus *Sorbus* to five hybridogenous genera (*Borkhausenia* = *Aria* × *Sorbus* × *Torminalis*, *Hedlundia* = *Aria* × *Sorbus*, *Karpatiosorbus* = *Aria* × *Torminalis*, *Majovskya* = *Aria* × *Chamaemespilus*, *Normeyera* = *Aria* × *Chamaemespilus* × *Sorbus*).

*Karpatiosorbus* has a hybrid origin from *Aria* (Pers.) Host × *Torminalis* Medik, including one sexual hybrid (*Karpatiosorbus* × *hybrida* (Borkh.) Sennikov & Kurtto) and 84 apomictic species (Sennikov and Kurtto, 2017). Among these 85 species, *Karpatiosorbus bristoliensis* (Wilmott) Sennikov & Kurtto is a shrub or small tree approximately 30 ft high and has restricted areas of distribution in Avon Gorge, England. It inhabits open limestone outcrops, slopes, scrub, open grassland, quarry edges, and ancient woodland plateau soils, primarily within the Leigh Woods ecosystem (Rich et al., 2022). Despite its ornamental value, this species has been assessed as ‘Endangered’ under criterion D (IUCN, 2001) and on the Global Red List owing to its small population size (Rich et al., 2022). Using six chloroplast fragments, Chester et al. (2007) revealed that *K. bristoliensis* and *Sorbus torminalis* (L.) Crantz shared an identical haplotype (haplotype B), while proposing that *K. bristoliensis* may represent a case of *in situ* speciation. Employing microsatellite analysis, Robertson et al. (2010) determined the triploid hybrid origin of *K. bristoliensis* with diploid *S. torminalis* as the maternal progenitor and tetraploid *S. eminens* E.F.Warb. as the paternal progenitor (Robertson et al., 2010). Previous studies utilizing organellar genome have provided novel insights into the genetic diversity and conservation of endangered species (Zhang et al., 2024). However, the limited genomic information available for this endangered species hinders our understanding of its genomic structure and genomic diversity. The organellar genomes assembled in this study will lay the foundation for conservation genomics in *K. bristoliensis*.

The mitochondrial genome has received increasing attention in the field of plant phylogenomics (Lin et al., 2025). This is because of the slow evolutionary rate of mitochondrial genes compared with nuclear genes. Unlike the chloroplast genome, plant mitochondrial genomes are large (66 kb to 18.99 Mb) (Skippington et al., 2015; Huang et al., 2024a), exhibit frequent recombination, and often incorporate foreign DNA via horizontal gene transfer (HGT) from chloroplasts and nuclear (Wang et al., 2025; Zhang et al., 2025). These features result in highly variable genome sizes and architectures across plant lineages (Gualberto and Newton, 2017; Wu et al., 2022; Lu et al., 2025). The plant mitochondrial genome harbors abundant repetitive sequences, including simple sequence repeats (SSRs), tandem repeats, and interspersed repeats. These repetitive elements serve as a foundation for the development of diverse molecular markers to investigate population genetic diversity (Morgante et al., 2002). Moreover, the high abundance of repeats contributes significantly to the complex structural rearrangements observed in mitochondrial genomes, such as recombination-mediated inversions, duplications, and the formation of multipartite architectures (Bi et al., 2022).

The availability of extensive chloroplast genome sequencing data for the genus *Sorbus* s.l. enables the utilization of these datasets to address unresolved phylogenetic questions within this taxonomic group. Furthermore, recent advances in long-read sequencing have resolved complex plant mitochondrial genome structures. Taking advantage on the previously released PacBio HiFi sequencing data of *Karpatiosorbus bristoliensis* in the NCBI database, we utilized these datasets to perform the first *de novo* assembly of both chloroplast and mitochondrial genomes for the hybrid genus *Karpatiosorbus*. Our main goals are to (1) assemble and characterize the first mitochondrial and plastid genomes of this species; (2) compare the mitochondrial genomes of *Sorbus* s.l. species; (3) explore repetitive sequences and RNA editing sites; and (4) reconstruct the maternal phylogenetic framework within *Sorbus* s.l. utilizing organellar genomes.

## Materials and methods

### Plant material, mitochondrial genome assembly and annotation

We obtained PacBio-HiFi data for *Karpatiosorbus bristoliensis* (ERR13245294) from the SRA (https://www.ncbi.nlm.nih.gov/sra/?term=ERR13245294). The whole-genome sequencing of *K. bristoliensis* revealed a total of 50.3G bases. For mitogenome assembly, we employed PMAT v2.1.2 (https://github.com/aiPGAB/PMAT2/releases) (Bi et al., 2024), an efficient plant mitogenome assembly toolkit using low-coverage HiFi sequencing data, to perform *de novo* assembly of the *K. bristoliensis* mitogenome with the command “PMAT autoMito -I -o -st -g -m”. For plastid genome assembly, the ptGAUL v1.0.5 pipeline (https://github.com/Bean061/ptgaul) (Zhou et al., 2023) was applied to extract and assemble the plastid genome from PacBio-HiFi long-read data with standard parameters. We employed Bandage (Wick et al., 2015) to visualize the graphical representation of the mitochondrial and plastid genomes produced by the PMAT and ptGAUL pipelines, respectively. Mitogenome annotation was performed via the online program Plant Mitochondrial Genome Annotator (PMGA) (http://47.96.249.172:16084/index.html), with the database set as 319 mitogenomes (Li et al., 2024). This database uses multiple sequence alignment to identify genes. The plastid genome annotation was performed via the online program CPGAVAS2 (http://47.96.249.172:16019/analyzer/home) (Shi et al., 2019), with *Sorbus helenae* (NC_068536.1) as the reference genome. Manual corrections were performed via Sequin software. We utilized OGDRAW (v.1.3.1) (https://chlorobox.mpimp-golm.mpg.de/ogdraw.html) to generate plastid genome and mitogenome maps.

### Analysis of repeat sequences

The prediction of simple sequence repeats (SSRs) was performed using the online program MISA-web (https://www.web-blast.ipk-gatersleben.de/misa/). The parameters of mono-, di-, tri-, tetra-, penta-, and hexanucleotides were set to 10, 5, 4, 3, 3, and 3, respectively. Tandem repeats within the five species were detected using Tandem Repeats Finder v4.09 (Benson, 1999) with default parameters. Dispersed repeats were identified using the online REPuter program (https://bibiserv.cebitec.uni-bielefeld.de/reputer) (Kurtz, 2001) with a minimal repeat size of 30 bp and a Hamming distance of 3.

### Homologous fragment analysis

we employed the online BLAST tool (https://blast.ncbi.nlm.nih.gov/Blast.cgi) to search highly similar sequences between the mitogenome and plastid genomes of *K. bristoliensis, M. alnifolia* (Siebold & Zucc.) Koehne, T. *glaberrima* (Gand.) Sennikov & Kurtto*, S. aucuparia* L., and *Sorbus aucuparia* subsp. *pohuashanensis* (Hance) McAll., respectively. The identification parameters for homologous sequences were set to ≥70% minimum similarity and ≤1e-5 E-value threshold.

### Detection of RNA editing events within *Sorbus* s.l.

We employed the online program PMGA (http://47.96.249.172:16084/deepredmt.html) to predict RNA editing events in the mitogenomes of *K. bristoliensis, M. alnifolia, T. glaberrima, S. aucuparia, and S. aucuparia subsp. pohuashanensis*. This online program uses Deepred-Mt (Edera et al., 2021), a novel deep convolutional neural network, to detect potential RNA editing events. The cut-off value was set to 0.5 for accurate prediction.

### Nucleotide diversity analysis

For plastid genome analyses, plastid genome sequences from a total of 59 species of *Sorbus* s.l. were downloaded. We used the command “cpstools Pi -d work_dir” from the CPStools package (Huang et al., 2024b) to calculate Pi values for the analyzed species. This package automatically extracts shared gene regions and intergenic sequences from GenBank-format files in work_dir, performs multiple-sequence alignments, and computes Pi values. For mitogenome analyses, we utilized PhyloSuite v1.2.3 (Zhang et al., 2020) to extract protein-coding genes from the five *Sorbus* s.l. species using default parameters. A total of 35 shared protein-coding genes were aligned using MAFFT (https://mafft.cbrc.jp/alignment/server/) (Katoh et al., 2018) with the default parameters. The nucleotide diversity (Pi) of each gene was subsequently calculated using DnaSP v6 (Rozas et al., 2017) with the option “compute variance of Pi”.

### Phylogenetic analysis

The mitochondrial phylogenomic tree was reconstructed via 14 mitochondrial genomes, including those of five *Sorbus* s.l. species (*Sorbus aucuparia* subsp. *pohuashanensis* ON478177, *Sorbus aucuparia* MT648825, *Micromeles alnifolia* PP506330, *Torminalis glaberrima* MT610102, and *Karpatiosorbus bristoliensis*), with *Rosa rugosa* (PQ474155) designated as the outgroup. We employed PhyloSuite v1.2.3 (Zhang et al., 2020) to extract protein-coding genes that were shared by the 14 species. Multiple sequence alignment was subsequently performed using MAFFT (https://mafft.cbrc.jp/alignment/server/) (Katoh et al., 2018) with default parameters. The aligned sequence trimming was performed using the trimAl program within the PhyloSuite v1.2.3 (Zhang et al., 2020) with the default (-automated1) parameter. We performed phylogenetic reconstruction using maximum likelihood method in IQ-TREE v2 (Minh et al., 2020), with 1,000 bootstrap replicates (-bb 1000) and 1,000 replicates for the SH-like approximate likelihood ratio test (SH-aLRT; -alrt 1000). The optimal nucleotide substitution model was determined using ModelFinder (Kalyaanamoorthy et al., 2017) in IQ-TREE v2, which identified K3Pu+F+R4 as the best-fit model based on the Bayesian Information Criterion (BIC).

A total of 65 plastomes, including those of 59 *Sorbus* s.l. species, were utilized for phylogenetic analyses (**Supplementary Table S1**). The whole plastid genomes were aligned with MAFFT (https://mafft.cbrc.jp/alignment/server/) (Katoh et al., 2018) with default parameter. Phylogenetic reconstruction employed identical software and parameters as above, with the TIM+F+R4 nucleotide substitution model specified according to the Bayesian Information Criterion (BIC)

### Collinearity analysis

We performed pairwise sequence alignments among five species of the *Sorbus* s.l genus using MUMmer v4.0.1 (Marçais et al., 2018), with the parameters set as: nucmer –maxmatch -c 100 -b 500 -l 50. Filtering of aligned sequences was performed with the following parameters: a minimum alignment identity of 90% and a minimum alignment length of 50 bp. Identification of collinear sequences and structural rearrangement events among mitochondrial genomes was conducted using SyRI v1.7.0 (Goel et al., 2019). Genome structural visualization was generated using Plotsr v1.1.0 (Goel and Schneeberger, 2022).

## Results

### General features of *Karpatiosorbus bristoliensis* mitogenome and plastome

**Figure 1:**
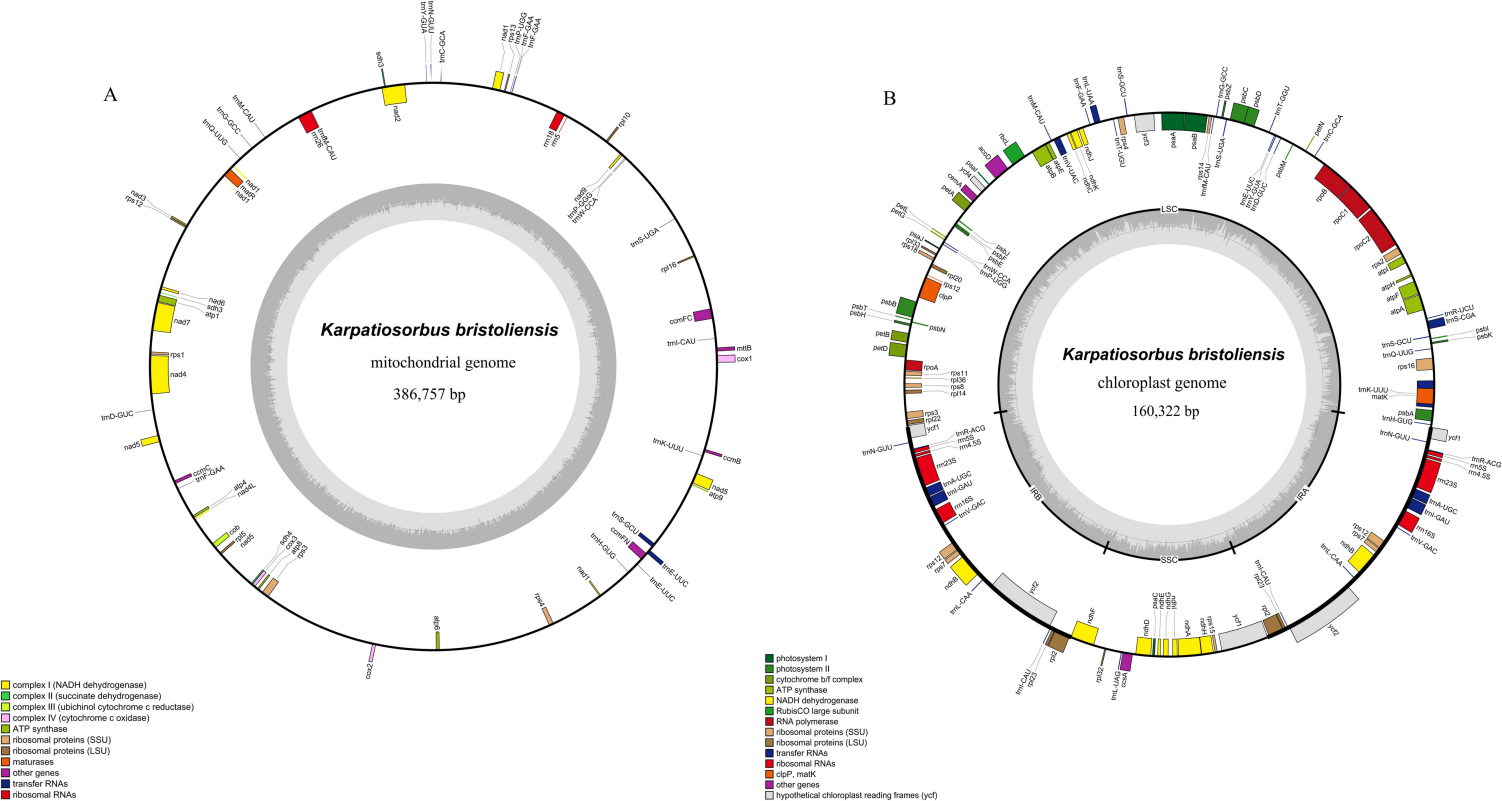
Mitochondrial and chloroplast genome diagram of *K*. *bristoliensis*. **(A)** mitogenome; **(B)** Plastome. Genes encoded on the forward strand are positioned on the outer circumference, while those on the reverse strand are located on the inner circumference. The gray inner ring represents the GC content.

We employed the autoMito program in PMAT to assemble the mitochondrial genome of *K. bristoliensis*. The average depth of the assembled results was 1408.7x (**Supplementary Figure S1**). The mitochondrial genome of *K. bristoliensis* was 386,757 bp in size. It encoded 55 unique genes, including 34 protein-coding genes, 18 tRNA genes and 3 rRNA genes (**Figure 1A**). The GC content was 45.3%. Three genes (*ccmFC, trnE-UUC, trnS-GCU*) had one intron, one gene (*nad4*) had three introns, and four genes (*nad1, nad2, nad5, nad7*) had four introns (**Supplementary Tables S2**, **S3**).

The *K. bristoliensis* plastid genome has structures identical to those of most angiosperm plants, consisting of one large single copy (LSC), one small single copy (SSC), and two inverted repeats (IRs) (**Figure 1B**). The total length of the plastid genome was 160,322 bp, including 88,182 bp, 19,306 bp and 26,417 bp in the LSC, SSC and IR regions, respectively. The GC content was 36.50%. The plastid genome encodes 75 protein-coding genes, 29 tRNA genes, four rRNA genes, and one pseudogene (*ycf1*Ψ) for a total of 108 unique genes. Among these genes, eight genes (*rps16, atpF, rpoC1, petB, petD, ndhB, rpl2, ndhA*) have one intron, and two genes (*ycf3, clpP*) possess two introns (**Supplementary Table S4**).

### Repeat sequence analyses

**Figure 2:**
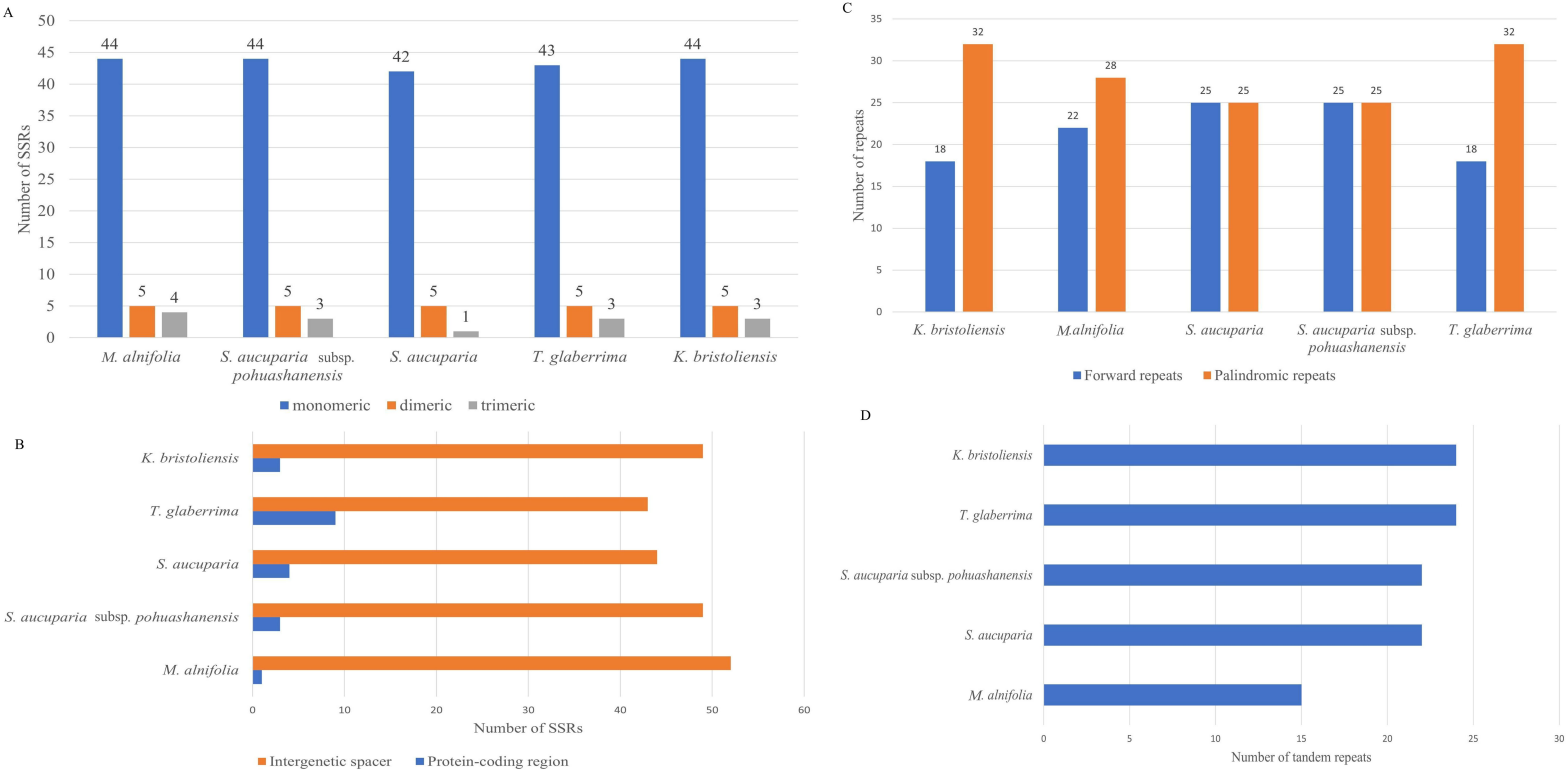
Distribution of repeat sequence in *Sorbus* s.l. mitogenome. **(A)** the number of SSRs in mitogenome; **(B)** distribution of SSRs in mitogenome; **(C)** the number of dispersed repeats; **(D)** the number of tandem repeats.

We detected 52, 53, 52, 48, and 51 simple sequence repeats (SSRs) in the mitochondrial genomes of *K. bristoliensis, M. alnifolia, S. aucuparia* subsp. *pohuashanensis, S. aucuparia*, and *T. glaberrima*, respectively (**Figure 2A**). All five species possess three types of SSRs: monomeric, dimeric, and trimeric repeats, with monomeric repeats being the most abundant. Notably, most SSRs were distributed in intergenic spacer regions, with fewer detected in protein-coding regions (**Supplementary Table S5**). Within protein-coding regions, SSRs primarily occurred in *nad1*, *nad2*, and *nad3* genes (**Figure 2B**). We identified 50 dispersed repeats in each species, consisting of forward and palindromic repeats (**Figure 2C**). Repeat sequence lengths ranged from 91–428 bp (*K. bristoliensis*), 88–2745 bp (*M. alnifolia*), 76–26,130 bp (*S. aucuparia* subsp. *pohuashanensis*), 86–6452 bp (*S. aucuparia*), and 80–438 bp (*T. glaberrima*) (**Figure 2C**). Notably, *S. aucuparia* contained two large palindromic repeats (26,130 bp and 24,730 bp; **Supplementary Table S6**). A total of 24, 15, 22, 22, and 24 tandem repeats were identified in the mitochondrial genomes of *K. bristoliensis, M. alnifolia, S. aucuparia* subsp*. pohuashanensis, S. aucuparia*, and *T. glaberrima*, respectively (**Figure 2D**) (**Supplementary Table S7**).

### Analysis of inter-organellar gene transfer events

Mitochondrial genome analyses revealed 14, 13, 12, 12, and 14 chloroplast-derived DNA fragments in *K. bristoliensis*, *M. alnifolia*, *T. glaberrima*, *S. aucuparia*, and *S. aucuparia* subsp. *pohuashanensis*, respectively (**Supplementary Table S8**). The lengths of these transferred sequences exhibited limited variation, ranging from 3,846 bp (*K. bristoliensis*), 3,834 bp (*M. alnifolia*), 3,830 bp (*T. glaberrima*), 3,755 bp (*S. aucuparia*), and 3,021 bp (*S. aucuparia* subsp. *pohuashanensis*). Notably, the largest individual transferred fragment spanned 1,874 bp. Notably, *K. bristoliensis*, *T. glaberrima*, and *S. aucuparia* subsp*. pohuashanensis* each contained five fully transferred tRNA genes (*trnW-CCA*, *trnD-GUC*, *trnH-GUG*, *trnN-GUU*, and *trnM-CAU*). In contrast, *M. alnifolia* and *S. aucuparia* exhibited four completely transferred tRNA genes (*trnW-CCA*, *trnH-GUG*, *trnD-GUC*, and *trnN-GTT*). Furthermore, partial transfer events involving protein-coding genes were detected, including fragments of *psbE*, *psbC*, *psaA*, *psbA*, and *atpA.*

### Prediction of RNA editing sites

**Figure 3:**
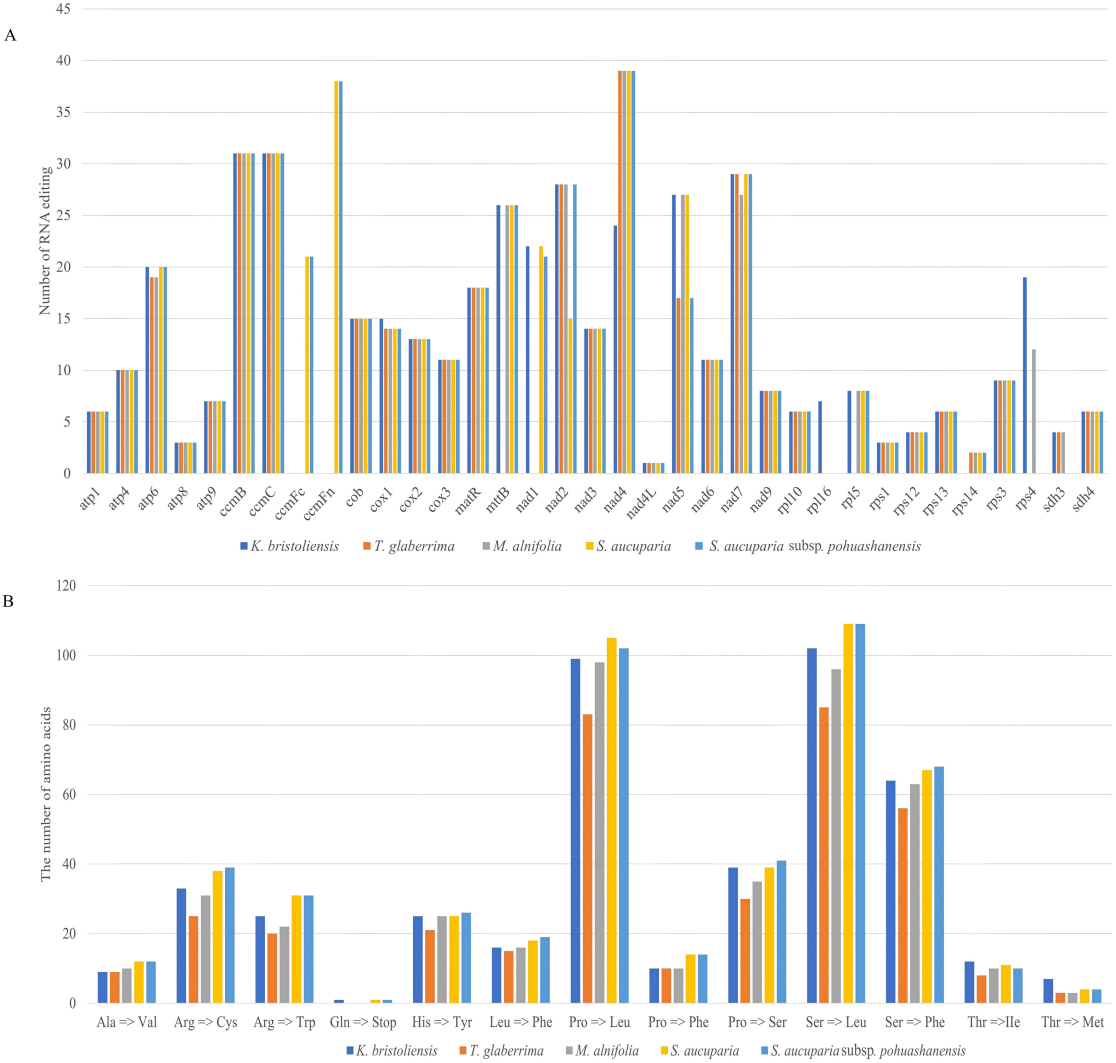
The number of RNA editing sites identified in the mitogenomes. **(A)** the number of RNA editing sites in *Sorbus* s.l. mitogenome; **(B)** number of types of RNA editing.

A total of 442, 403, 474, 463, and 352 RNA editing sites were identified in the mitochondrial genomes of *K. bristoliensis*, *M. alnifolia*, *S. aucuparia*, *S. aucuparia* subsp. *pohuashanensis*, and *T. glaberrima*, respectively (**Figure 3A**). Comparative analysis revealed that three taxa (*K. bristoliensis*, *S. aucuparia*, and *S. aucuparia* subsp. *pohuashanensis*) each contained RNA editing modifications in 32 protein-coding genes, while *M. alnifolia* and *T. glaberrima* possessed 31 and 28 edited genes, respectively. Notably, *nad4L* exhibited the lowest frequency of RNA editing events among all protein-coding genes across the five species, each containing a single editing site. The *nad4* gene demonstrated the highest RNA editing activity in four mitochondrial genomes (*M. alnifolia, S. aucuparia, S. aucuparia* subsp. *pohuashanensis*, and *T. glaberrima*), with 39 editing sites identified in *M. alnifolia*. In contrast, the *K. bristoliensis* mitochondrial genome showed unique editing patterns, where *ccmB* and *ccmC* collectively contained the highest number of RNA editing sites (31 total), representing a distinct regulatory feature in this species. All RNA editing sites belong to C to U. Notably, the start codons of *cox1, nad1, rpl16*, and *rps4* in the mitochondrial genome *of K. bristoliensis*, through RNA editing, have changed from ACG to AUG. Additionally, RNA editing has contributed to stop codons for *atp6* in the mitochondrial genomes of *K. bristoliensis*, *S. aucuparia*, and *S. aucuparia* subsp*. pohuashanensis* (**Supplementary Table S9**). The most frequent amino acid substitution was serine-to-leucine, except in *M. alnifolia* where proline-to-leucine predominated (**Figure 3B**).

### Nucleotide diversity analysis

**Figure 4:**
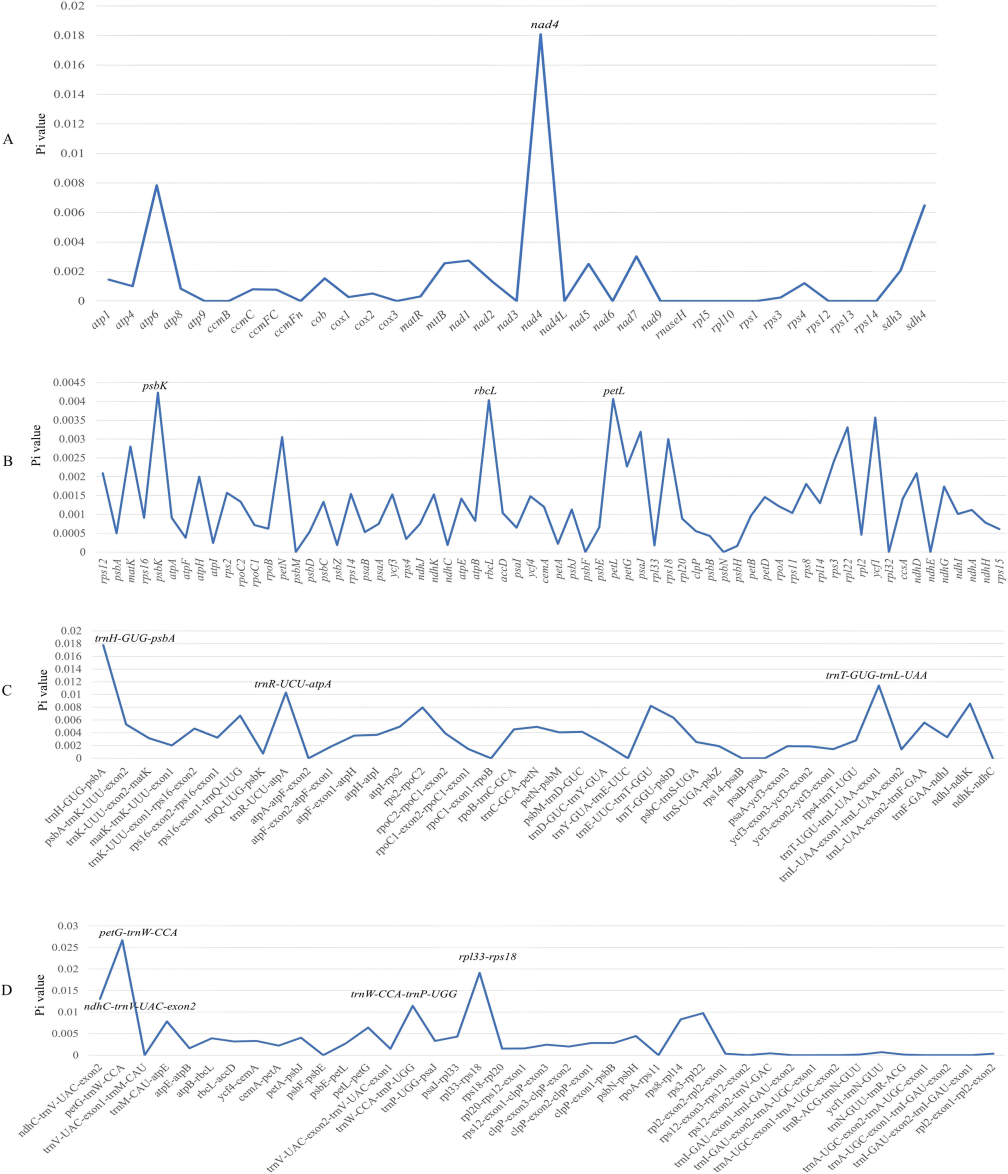
Nucleotide diversity analysis of *Sorbus* s.l. species. **(A)** mitogenome; **(B–D)** plastome.

Nucleotide diversity analysis was performed using DnaSP v6.0. For mitochondrial genomes, the Pi values ranged from 0 to 0.01808, with the *nad4* gene exhibiting the highest diversity (0.01808) (**Figure 4A**). In plastid genomes, the Pi values showed greater conservation, spanning from 0 to 0.02672 (*petG-trnW-CCA*) (**Figures 4B–D**). Comparative analysis identified seven hypervariable regions as potential molecular markers. Seven regions with relatively high Pi values may be designed for DNA barcodes, including *trnH-GUG-psbA*, *trnT-GUG-trnL-UAA*, *petG-trnW-CCA*, *ndhC-trnV-UAC-exon2*, *trnW-CCA-trnP-UGG*, *rpl33-rps18*.

### Phylogenomic analyses

**Figure 5:**
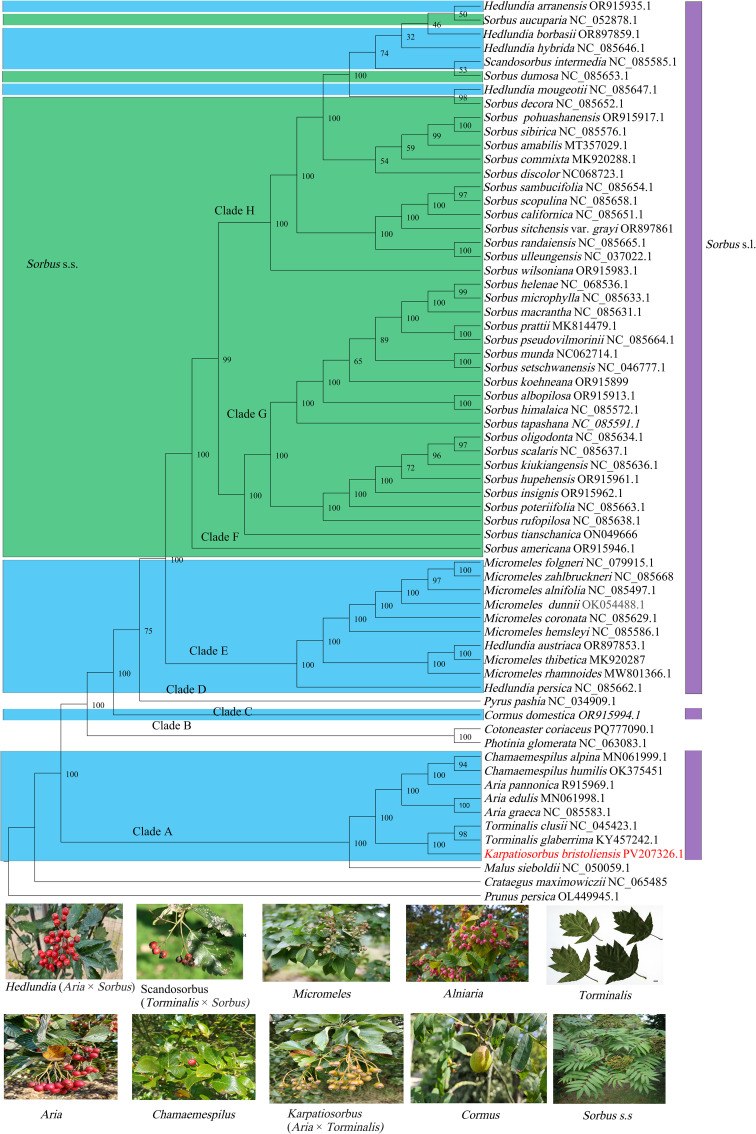
The maximum likelihood phylogeny reconstructed with IQ-TREE v2 displays bootstrap values on branches. Colors in the figure denote *Sorbus* s.l. lineages, with green designating *Sorbus* s.s. clades. Botanical images beneath the phylogeny represent constituent genera of *Sorbus* s.l. included in this study, with parenthetical notation indicating documented hybrid origins.

We recovered a plastome-based phylogeny of *Sorbus* s.l., which included an ingroup of 59 accessions (**Figure 5**). Our plastome-based phylogeny yielded strong bootstrap values (> 90%) for the vast majority of branches. With robust node support, we recovered eight major clades (A, B, C, D, E, F, G, H). Among these clades, *Sorbus* s.l. was dispersed in six clades. Clade A contains four genera (*Chamaemespilus*, *Aria*, *Torminalis*, and *Karpatiosorbus*) of *Sorbus* s.l. *Karpatiosorbus*, which are closely phylogenetically related to *Torminalis*. The genus *Aria* was found to be nonmonophyletic, with one species embedded in the genus *Chamaemespilus*. Clade C contains one species, *Cormus domestica.* Clade E corresponds to the genera *Micromeles* and *Hedlundia*. *Micromeles* are not monophyletic, with *Hedlundia* embedded in them. Clades F-H correspond to *Sorbus* s.s. and are expected to include the hybrid-origin genus *Hedlundia* and *Scandosorbus*.

**Figure 6:**
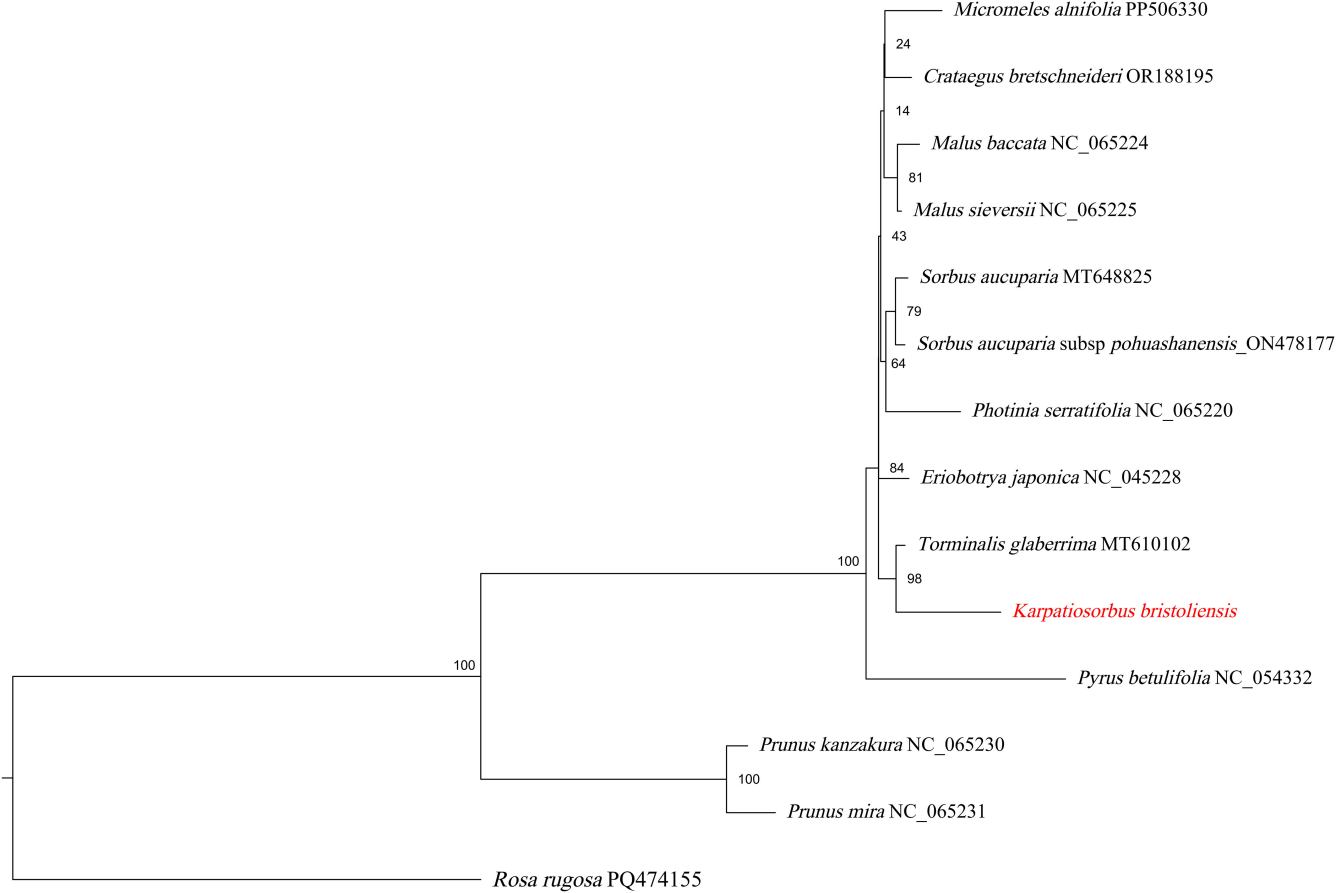
ML phylogeny (IQ-TREE v2; 34 shared protein-coding genes) showing bootstrap values on branches.

To better understand the mitochondrial genome evolution of *Sorbus* s.l., we also utilized the maximum likelihood method to construct mitochondrial genome-based phylogenetic trees based on 35 shared protein-coding genes (**Figure 6**). The *Prunus* genus was used as the outgroup. Most nodes of the phylogenetic tree presented relatively lower bootstrap values than those of the plastome-based phylogenetic tree did. Consistent with the plastome-based phylogeny, the genus *Karpatiosorbus* is a sister to *Torminalis*.

### Analysis of mitogenome collinearity and structural rearrangement

**Figure 7:**
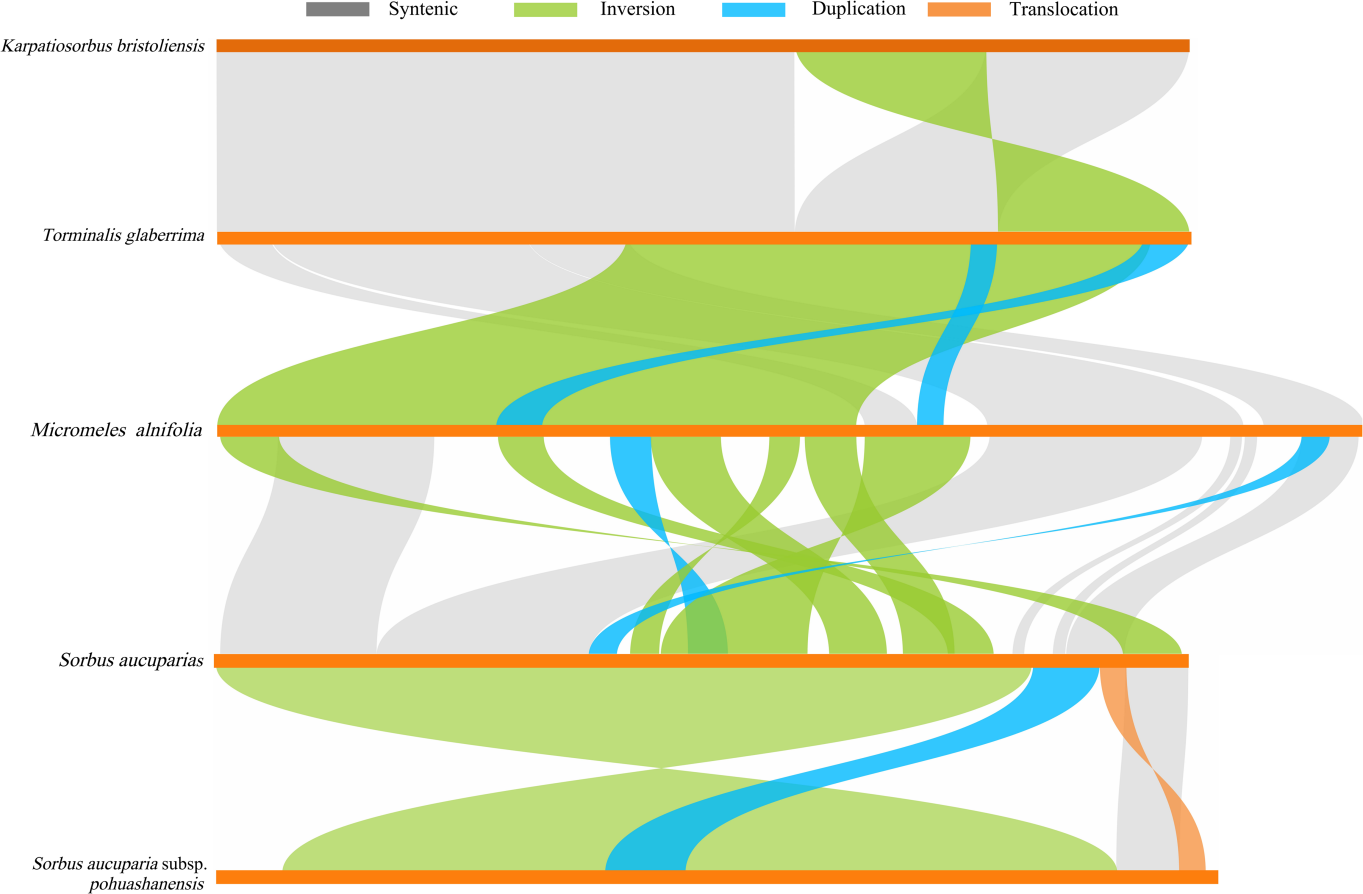
Collinearity Analysis of Mitochondrial Genomes in Five *Sorbus* s.l. Species. The gray arcs represent structurally homologous sequences, while other colors indicate structural rearrangements: inversions (green), transpositions (blue), and duplications (orange).

Analysis of mitochondrial genome sequences and structures across five *Sorbus* species revealed extensive collinear regions, encompassing structural homology, translocations, and inversions (**Figure 7**). The longest sequence-similarity segments between species pairs were: 84,781 bp (*M. alnifolia* vs *S. aucuparia*), 97,089 bp (*S. aucuparia* vs *S. aucuparia* subsp. *pohuashanensis*), 229,635 bp (*K. bristoliensis* vs *T. glaberrima*), and 101,625 bp (*T. glaberrima* vs *M. alnifolia*). The highest degree of syntenic segment was observed between *K. bristoliensis* and *T. glaberrima* (310,482 bp), accounting for 80.28% of the *K. bristoliensis* mitochondrial genome length. Additionally, a 76,099 bp inversion structure was identified in the *K. bristoliensis* and *T. glaberrima* (**Supplementary Table S10**).

## Discussion

### Structure and size variation of chloroplast and mitochondrial genomes

This study reports the first complete mitochondrial and plastid genome assemblies for the endangered tree species *K. bristoliensis*. The circular mitochondrial genome spans 386,757 bp, demonstrating near-identical size conservation (1 bp difference) with its maternal progenitor *Torminalis glaberrima* (386,756 bp), consistent with its hybrid origin (*Aria* × *Torminalis*). The chloroplast genome of *K. bristoliensis* is 68 bp smaller than that of *Torminalis glaberrima*. Compared with those of other species within *Sorbus* s.l., the mitochondrial genome of *K. bristoliensis* was smaller than those of *M. alnifolia* (45,5361 bp) and *S. aucuparia* subsp. *pohuashanensis* (396,857 bp) but larger than that of *Sorbus aucuparia* (384,977 bp). Previous studies have reported that the most minor mitochondrial genome in Rosaceae is *Taihangia rupestris* var. *ciliata* T.T.Yu & C.L.Li, with a size of 265,633 bp (Li et al., 2025), and the largest one is *Prunus mume* (Siebold) Siebold & Zucc. (535,727 bp) (Sun et al., 2022). The mitochondrial genome size of *Sorbus* s.s. falls within the range of mitochondrial genome size variation observed in Rosaceae. The mitochondrial genome of *K. bristoliensis* exhibited a GC content of 45.3%, aligning closely with the conserved genomic composition observed across Rosaceae mitogenomes (range: 43.31%–45.62%); (Lu et al., 2023).

### Characteristics of repetitive sequences in the mitochondrial genome of *Sorbus* s.l.

SSRs exhibit high polymorphism and codominant inheritance, thus frequently used in population genetics studies (Hu et al., 2019). This study identified a large number of SSRs within the mitochondrial genome of *Sorbus* s.l., which exhibit potential utility for population genetic investigations. The number of SSRs in the mitochondrial genome of *Sorbus* s.l. is comparable to that observed in other Rosaceae species such as *Rubus idaeus* L (Zhang et al., 2025), yet demonstrates a lower frequency compared to members of the *Malus* genus within the same family (Wang et al., 2024). Consistent with previous findings, monomeric repeats predominated among the three SSR motif categories, exhibiting the highest numerical abundance (Niu et al., 2024; Sun et al., 2024). Tandem repeats may contribute to mitochondrial genome size expansion, particularly in species with exceptionally large genomes (Wynn and Christensen, 2019). Some tandem repeats exhibit species-specific amplification, serving as molecular markers for phylogenetic studies. A total of 107 tandem repeats were detected among the five species, which will be helpful for future studies. Dispersed repeats are nonadjacent homologous sequences ranging from tens to thousands of base pairs and are often distributed throughout the mitochondrial genome. These repeats facilitate homologous recombination, a key driver of structural rearrangements such as inversions, duplications, and the generation of subgenomic circles (Wu and Sloan, 2019). Previous studies have demonstrated prevalent mitochondrial genome rearrangements in Rosaceae species, as exemplified by members of the genus *Malus* (Wang et al., 2024) and *Fragaria* (Fan et al., 2022). Notably, one large palindromic repeat (26,130 bp) and forward repeat (24,730 bp) were detected in the mitochondrial genome of *S. aucuparia*. In addition, the *S. aucuparia* subsp. *pohuashanensis* mitogenome also presented one forward repeat exceeding 6,000 bp (6452 bp). Whether these large repeats are involved in mitochondrial genome rearrangement and other structural evolution remains to be further studied.

### Gene transfer between mitochondrial and chloroplast genomes

Horizontal gene transfer (HGT) from chloroplasts to mitochondrial genomes represents a notable phenomenon in plant organelle evolution, reflecting complex inter-organellar genomic interactions (Yang et al., 2022). Although chloroplast-to-mitochondrion gene transfers occur less frequently than chloroplast-to-nuclear transfers do, accumulating evidence reveals their evolutionary significance in mitogenome evolution (Hong et al., 2021). In this study, we identified some chloroplast fragments transferred to the mitochondrial genome, with sizes ranging from 3,021 bp to 3,856 bp. Compared with previous studies in other Rosaceae, such as *Rubus idaeus* L. (46,456 bp transferred from chloroplasts to mitochondria) (Zhang et al., 2025), we found that homologous fragment transfer between the plastome and mitogenome in *Sorbus* s.l. was relatively rare. These findings indicate that the mitochondrial and chloroplast genomes of *Sorbus* s.l. are relatively highly conserved. Consistent with previous studies (Wang et al., 2007), we also found that the most frequently transferred genes were tRNAs, with a total of five tRNA genes (*trnW-CCA, trnD-GUC, trnH-GUG, trnN-GUU, and trnM-CAU*) shared by the plastid genome and the mitochondrial genome.

### RNA editing in *Sorbus* s.l. mitochondrial genome

RNA editing is frequently reported in plant mitochondrial genomes (Liu et al., 2024). It is a crucial posttranscriptional modification process that plays a crucial role in controlling gene expression and functionality (Liu et al., 2020). At present, the common type of RNA editing is C–U editing, and G-to-C and T-to-A editing can also occur (Liu et al., 2024; Ichinose and Sugita, 2016; Yang et al., 2022). In this study, all RNA editing sites in *Sorbus* s.l. were associated with C–U editing. The number of RNA editing sites differed greatly in *Sorbus* s.l., ranging from 352 (*T. glaberrima*) to 463 (*S. aucuparia subsp. pohuashanensis*). Compared with those in other Rosaceae species, the number of RNA editing sites in *Sorbus* s.l. species was greater than that in *Prunus pedunculata* (262) (Liu et al., 2023) but less than that in *Rubus chingii* var. *suavissimus* (492) (Shi et al., 2024) and *Taihangia rupestris* var. *ciliata* (470) (Li et al., 2025). Overall, RNA editing sites exhibit significant variations across different species and even within the same genus, such as *S. aucuparia* subsp. *pohuashanensis* and *S. aucuparia*. Notably, the RNA editing sites appear to exhibit a gene preference, with editing events occurring most frequently within the NADH dehydrogenase genes. Similar results have been reported in other species, such as *Prunus pedunculata* (Pall.) Maxim. (Liu et al., 2023) and *Gelsemium elegans* (Gardner & Champ.) Benth (You et al., 2023). We also observed that RNA editing influenced the start and termination codons of protein-coding genes. For example, the start codons of *cox1, nad1, rpl16, and rps4* changed from ACG to AUG through RNA editing, whereas RNA editing led to the termination codons of *atp6*. RNA editing events that alter initiation and termination codons are frequently observed, as exemplified by modifications to the *nad1* initiation codon and *ccmFc* termination codon in the mitochondrial genome of *Garcinia mangostana* L (Wee et al., 2022).

### Capture of polymorphic loci

The mitochondrial and chloroplast genomes harbor a substantial number of polymorphic sequences that can be developed as DNA barcodes for phylogenetic and population genetic studies (Li and Wei, 2022). This study analyzed the mitochondrial genomes of five *Sorbus* s.l. species and revealed that the *nad4* gene presented the greatest number of nucleotide polymorphisms. These findings suggest its potential utility as an effective DNA barcode for investigating phylogenetic relationships within *Sorbus* s.l. Through comparative analysis of chloroplast genomes, we identified six plastid gene regions exhibiting high genetic polymorphism, which could serve as chloroplast DNA barcodes for investigating phylogenetic relationships within *Sorbus* s.l. Furthermore, the *ycf1* gene, conventionally employed as a universal barcode for angiosperms (Dong et al., 2015), demonstrated lower nucleotide polymorphism in this study.

### Maternal lineage phylogenetic analysis of the *Sorbus* s.l.

In this study, we provide a high-resolution maternal genetic framework for *Sorbus* s.l. *Aria pannonica* (Kárpáti) Sennikov & Kurtto, which is a hybrid-origin species with a ploidy level of triploid (Somlyay and Sennikov, 2015). *A. pannonica* originated from intragenus hybridization because of its morphological similarity to *A. nivea* Host and *A. graeca* (Spach) M.Roem (Somlyay and Sennikov, 2015). However, *A. pannonica* is more closely related to the genus *Chamaemespilus* in our plastome-based phylogeny. Therefore, we inferred that the female parent of this hybrid species may have originated from *Chamaemespilus*. This fact indicated that *Aria pannonica* should treat as the member of *Majovskya*. The genus *Karpatiosorbus* is thought to originate from the hybridization of *Aria* and *Torminalis* (Pellicer et al., 2012). Both our mitogenome- and platome-based phylogenetic data supported that *Karpatiosorbus* and *Torminalis* clustered into one branch. These findings indicate that the female parent of this genus is *Torminalis*. The genus *Hedlundia* contains one sexual hybrid and 39 apomictic species and originates from crosses between various species of *Aria* and *Sorbus* s.l (Sennikov and Kurtto, 2017). The genus *Hedlundia* is separated into two clades: one clade is closely related to *Micromeles*, and the other clade is embedded in *Sorbus* s.s, indicating the complex origin of the hybrid genus. Sennikov and Kurtto (2017) proposed the establishment of the new nothogenus *Sorbomeles* to accommodate hybrids of *Sorbus* and *Micromeles* origin. In the present study, molecular evidence suggests *Micromeles* likely served as the maternal progenitor of *Hedlundia austriaca* (Beck) Sennikov & Kurtto and *H. persica* (Hedl.) Mezhenskyj. Consequently, these two taxa are hereby formally proposed for reclassification into the hybrid genus ×*Sorbomeles.* A previous study indicated that hybridization between *Aria* and *Sorbus* s.s. contributed to the genus *Micromeles*. Both our results and those of previous plastome-based studies support that *Micromeles* is a sister to *Sorbus* s.s., whereas this is inconsistent with nuclear phylogenetic analyses (Zhang et al., 2023). Therefore, hybridization occurs between *Micromeles* and *Sorbus* s.s. *Scandosorbus intermedia* (Ehrh.) Sennikov originates from hybridization among *Sorbus* s.s., *Aria*, and *Torminalis* (Pellicer et al., 2012). It involves a complex species formation process. In this study, *S. intermedia* was a sister to *S. dumosa*. These findings indicate that the female parent of this species is *S. dumosa* House or a related species.

Phylogenetic analyses have consistently recovered *Sorbus* s.s. as a monophyletic clade distinct from other simple-leaved genera and the compound-leaved genus *Cormus* in previous studies (Campbell et al., 2007; Zheng and Zhang, 2007; Potter et al., 2007; Lo and Donoghue, 2012). This limitation arises from inadequate sampling in prior studies, which excluded phylogenetically critical hybrid-origin taxa. However, our current phylogenomic reconstruction reveals that *Hedlundia* and *Scandosorbus* (simple-leaved hybrid-origin genera) nests within *Sorbus* s.s., thereby rendering the latter non-monophyletic. This phylogenetic incongruence results from the hybrid origin of *Hedlundia* and *Scandosorbus*, which inherited its maternal genome from ancestral *Sorbus* s.s. lineages. *Sorbus* s.s. is one of the most typical examples of taxonomic complexity arising from the combined effects of hybridization, polyploidy and apomixis in the Rosaceae (Robertson et al., 2010). Previous research has demonstrated that both insect-pollinated and bird-pollinated are present in the genus *Sorbus* s.s.; moreover, mammals also play an important role in the spread of *Sorbus* s.s. seeds (Raspé et al., 2000; Bednorz et al., 2006). Some species of this genus are self-incompatible (Raspé et al., 2000). These factors strongly increase the frequency of interspecific hybridization in *Sorbus* s.s. as well as hybrid with other genera. Therefore, elucidating taxonomic challenges in *Sorbus* s.s requires resolving hybridization patterns and clarifying phylogenetic placements of hybrid-origin taxa within the genus.

### Frequent structural rearrangements of mitogenome

Mitochondrial genome structural complexity arises from extensive repetitive sequences and frequent structural rearrangements. Such mitochondrial genome rearrangements have also been frequently reported in other genera of Rosaceae, notably *Prunus* (Zhai et al., 2025) and *Malus* (Wang et al., 2024). This study revealed that *K. bristoliensis* shares substantial homologous segments with *T. glaberrima* (species from its maternal genus), while exhibiting limited homology with phylogenetically distant genera. This pattern aligns with their close phylogenetic origin and the hybrid origin of *Karpatiosorbus*. Despite the extensive shared homology, a 76,099 bp inversion structure was identified between their mitochondrial genomes, suggesting incipient mitogenomic divergence between the two genera.

## Conclusions

In this study, we reported the first complete mitochondrial and plastid genomes of the endangered tree *Karpatiosorbus bristoliensis* utilizing PacBio-HiFi long reads. *K. bristoliensis* mitochondrial and plastid genomes consists of one circular chromosome structures with the length of 386,757 bp bp and 160,322 bp, respectively. We analyzed repeat sequences, RNA editing, inter-organellar gene transfer, and pi value in the mitochondrial genome of *Sorbus* s.l., enriching our knowledge of mitochondrial genome evolution of this genus. We also conducted phylogenetic analyses within *Sorbus* s.l. utilizing mitochondrial and plastid genome. We emphasize that resolving hybridization dynamics in *Sorbus* s.l. is critical for achieving a taxonomically coherent delineation of the genus. Overall, our study provided a high-resolution maternal framework for *Sorbus* s.l.

## Data Availability

The mitochondrial and plastid genome have been deposited in GenBank under the accession numbers: PV207325 and PV207326.

## Supplementary material

Supplementary Figure 1 Bandage visual mitochondrial genome assembly map.
